# Constructing healthcare services markets: networks, brokers and the China-England engagement

**DOI:** 10.1186/s12992-022-00892-8

**Published:** 2022-12-09

**Authors:** Brian Salter, Yiming Dong, Benjamin M. Hunter

**Affiliations:** 1 Department of Political Economy, King’s College London, London, UK; 2 Department of International Development, King’s College London, London, UK; 3 Department of International Development, University of Sussex, Brighton, UK

**Keywords:** International healthcare services markets, China healthcare system, England healthcare system, Brokerage, Networks, Medical tourism

## Abstract

**Background:**

Healthcare services is an expanding international market with which national healthcare systems engage, and from which they benefit, to greater and lesser degrees. This study examines the case of the China-England engagement in healthcare services as a vehicle for illuminating the way in which such market relationships are constructed.

**Findings:**

China and England have different approaches to the international healthcare services market. Aware of the knowledge and technology gaps between itself and the leading capitalist nations of the West in healthcare, as in other sectors, the Chinese leadership has encouraged a variety of international engagements to facilitate the bridging of these gaps including accessing new supply and demand relationships in international markets. These engagements are situated within an approach to health system development based on establishing broad policy directions, allowing a degree of local innovation, initiating and evaluating pilot studies, and promulgating new programmatic frameworks at central and local levels. The assumption is that the new knowledge and technologies are integrated into this approach and implemented under the guidance of Chinese experts and leaders. England’s healthcare system has the knowledge resources to provide the supply to meet at least some of the China demand but has yet to develop fully the means to enable an efficient market response, though such economic engagement is supported by the UK’s trade related departments of state. As a result, the development of China-England commercial relationships in patient care, professional education and hospital and healthcare service development has been led largely by high status NHS Trusts and private sector organisations with the entrepreneurial capacity to exploit their market position. Drawing on their established international clinicians and commercial teams with experience of domestic private sector provision, these institutions have built trust-based collaborations sufficiently robust to facilitate demand-supply relationships in the international healthcare services market. Often key to the development of relations required to make commercial exchange feasible and practicable are a range of international brokers with the skills and capacity to provide the necessary linkage with individual healthcare consumers and institutional clients in China. Integral to the broker role, and often supplied by the broker itself, are the communication technologies of telemedicine to enable the interaction between consumer and healthcare provider, be this in patient care, professional education or healthcare service development.

**Conclusions:**

Although England’s healthcare system has the knowledge required to respond to China’s market demand and such economic engagement is supported and actively encouraged by the UK’s trade related departments of state, the response is constrained by multiple domestic demands on its resources and by the limits of the NHS approach to marketisation in healthcare.

## Introduction

National healthcare systems interact with each other within an increasingly integrated and marketized global health sector. In 2018 its total value expressed as public and private health expenditure combined was US$ 8452 billion with a compound annual growth rate (CAGR) since 2014 of 7% and a projected value in 2022 of US$ 11,910 billion ([112]: 3); [10, 24, 25]). By 2030 global per capita spending on health is expected to increase 50% [28]. Such growth is the result of sustained and expanding demand, on the one hand, and the construction of innovative modes of delivering the healthcare supply through the exploitation of new business models, sources of finance and communication technologies, on the other. And it is in the healthcare services sector of the market, rather than the pharmaceutical drugs and medical equipment sectors, that the most change is expected to occur. Accounting for 79% of the total value of the market in 2018 with the other sectors constituting the remaining 21% [10], the healthcare services sector is expected to have declined by 6% under the impact of Covid 19 in 2020 and then to recover and grow at a CAGR of 7% from 2021 and reach US$ 9725.4 billion in 2023 [11].

Some national healthcare systems engage with this expanding international market more than others with middle-income countries predicted to be the drivers of future growth. Propelled by the needs and rising expectations of an increasingly affluent middle class, countries such as China, India and Brazil are fuelling a rising demand both for healthcare services themselves and for the new market mechanisms which can ensure their prompt delivery ([23, 54] and 2016b [62];). For their part, several high- and middle- income countries are looking to provide services to meet these needs. The USA has long stood out amongst health services exporters, accounting for 24.4% of the total value of global healthcare exports in 2010 [58] - a reflection of the success of its universities and healthcare providers in building international reputations and portfolios in this sector [69, 89]. While several middle-income countries have become prominent in the supply of services to international medical travellers, a number of high-income countries are looking instead to opportunities in other export markets, for example education and training, clinical service development and infrastructure. For example, Japan’s 2013 Revitalization Strategy identified healthcare as one of four priority areas for the national economy, aiming to ‘promote global deployment of Japanese medical technologies and services’ ([35], p. 95); that same year the United Kingdom (UK) Government launched Healthcare UK with a similar mission.

In this paper we analyse the growth and differentiation of international markets for advisory services and knowledge resources designed to profit from the rapid expansion of public and private healthcare infrastructure in emerging economy countries. For market change on this scale in a sector as complex as healthcare services, new means are necessary to facilitate a relationship between the rising demand in one healthcare system and the supply of expertise, technology and investment in another. Such adaptive capacity does not appear automatically but has to be constructed through domestic investment and policy, public and private networks and brokerage, or, most likely, a combination of both. Situated within this arrangement, the skills to develop the international infrastructures of new demand-supply relationships themselves constitute a valuable resource with their own market value. Healthcare market construction through networks and brokerage has its own price. The degree to which that infrastructure price should be paid by the public purse and/or the private mechanisms of the market is an important question for a state’s healthcare system policy to address.

The empirical focus of the analysis is the engagement between the healthcare systems of China and England, countries which occupy contrasting positions in the international healthcare services market. On the one hand, China is an established and active player in that market as a result of the demand generated by its expanding middle class and ageing population, state support for the use of private healthcare markets, and the limited domestic supply of skills and expertise historically available to meet that demand. Significantly, China’s contribution to the global healthcare services market is predicted to increase by US$ 657 billion between 2018 and 2022 – the largest single contributor to growth and an impressive 23% of the global total [8]. On the other, England’s healthcare system has the policy ambition, skills and expertise to respond to that demand and has shown the policy ambition to make it happen [41]. Given this situation, how can we best understand the way in which fresh demand-supply relationships in the international healthcare services market may be facilitated through the entrepreneurial activities of institutions, networks and brokers?

The paper begins with a discussion of the insights provided by the literature on global markets in health-related services into how new market relationships are constructed and secured. Secondly, it examines the extent to which the healthcare systems of China and England are prepared to engage with the international market. What is their ideological and policy stance regarding the use of national and international market mechanisms in the delivery of their healthcare services? Thirdly, and informed by the earlier discussion, it provides a detailed profile of the nature of these market relationships in the case of the China-England engagement. What services are supplied, what institutions are involved and where do brokers fit into the market profile? Fourthly, the paper explores the role of networks and brokers in the construction of the conditions necessary for such a market profile to emerge, the importance of social trust to that process, and the contribution of state organisations. Finally, the paper reflects on the policy implications of its findings.

### Constructing healthcare markets

Studies of the growth of global markets for health-related services have tended to focus on the most visible services: those that involve large-scale movements of people such as cross-border travel to access healthcare services (sometimes referred to as ‘medical tourism’) [4, 15, 21, 22, 78], or the migration of health workers across borders [3, 51, 115]. Only a small number of studies have begun to document the wider range of services that are being traded across borders [63, 98], such as advisory services, education and training services, and branding and accreditation services, and which are the focus of our discussion in later sections. These services have arisen as the expansion of global demand for healthcare has in turn stimulated demand for medical and managerial knowledge that is expected to produce healthcare facilities of an ‘international’ standard and which can compete for domestic, and perhaps international, healthcare consumers. It is a market that has been promoted by the consultancy arms of companies for some time [6]. In China, for example, consultancy companies such as PriceWaterhouseCoopers China and Deloitte China have been active for many years providing audit, tax, financial advisory services, consulting services and enterprise risk services for health care providers, including hospitals, professional service providers, outpatient facilities and long term care (see eg [26, 84]).

More recently, hospitals in high-income countries, private and public [64], faced with budget constraints and with growing competition for their international healthcare consumers and falling numbers of travellers from Middle Eastern countries [82], have sought to capitalise on these developments. Encouraged by their national governments, they have commercialised their knowledge and reputation to engage in the cross-border provision of advisory services. This supply-side sensitivity to the opportunities available in the international healthcare services market finds expression in the construction of new business and organisational models to enable responsiveness to consumer demand through improved delivery. Supply-side market innovation is apparent, for example, in a continuing stream of mergers, acquisitions and partnering not only horizontally between healthcare providers but also vertically where non-healthcare companies searching for alternative revenue sources invest in what they see as the opportunities of the global healthcare services market ([23]: 9). This latter development can be seen as the latest phase of the ‘financialisation’ of healthcare services [47].

In addition to these general perspectives, two themes from the existing literature on global healthcare markets inform the analysis employed in this paper. First, there is the work on the policy trajectories that see states and regions emerge as buyers or suppliers of health-related services in global markets. Scholars have shown how governments in countries including India [85, 101], Malaysia and Singapore [14], Philippines [66], Turkey [63, 116], and South Korea and the UK [63, 64] have pursued export roles in global healthcare markets as part of an economic strategy to boost growth, using a combination of deregulation and investment/subsidy to expand forms of healthcare or education provision that can feed into global circuits of users and workers. Governments keen to access foreign revenue are supported by an enabling infrastructure of affordable travel, brokerage agencies providing facilitating support (see below), internet-based advertising and information, internationally recognised certifications and accreditations, and internationally portable health insurance [38]. Such activities see states and their social sectors situated within competitive global environments [63, 64]; as we show later in this article, this necessitates strategies for leading institutions to distinguish themselves from competitors in these environments.

Second, the research on the role of networks and brokers in mediating cross-border flows provides insights into the social dimensions of new market formation [65]. Networks are central to the construction of markets between healthcare systems because in market conditions where demand is uncertain and complex they have the advantage of enabling institutions to use social mechanisms for ‘adapting, coordinating and safeguarding exchanges’ through the creation and maintenance of social trust ([49]: 913). Reinforced by frequent interaction and the regular diffusion of values, norms and information, networks establish a form of ‘structural embeddedness’ which can both facilitate exchange and incorporate the reputational incentives and sanctions necessary to ensure compliance with the rules of the network [36]. In China such networks are described as ‘guanxi’ where reciprocity is a common feature of business interactions [114]. At the international level, cross-border networks are not purely self-constituting but depend on the help of national-territorially based networks – including the regulatory, financial, and infrastructural powers of the state.

Often supporting the integration of networks in the formation of market relations is the brokerage of demand-supply relationships by a mediating agency (Stovel and Shaw, 2014). Professional broker firms are common in service sectors characterised by complex systems of information and uncertainty such as finance [31], health insurance [50] and real estate [88]. In the case of the healthcare services market, the value of the brokerage activity derives from the provision of the link between the demand for and supply of expert knowledge [46]. Although such agencies are widely acknowledged in the study of cross-border travel for healthcare and for health work (see for example [46, 59]), their role in the trading of other services is not and forms an important focus in the research reported here.

### Healthcare systems and market construction: the case of China and England

Initiatives by states designed, in part, to enable their healthcare systems to respond to international healthcare consumer demand and to compete effectively in the international healthcare services market are of course dependent on, and shaped by, local political conditions. In the case of China and England, these conditions have produced different approaches to the use of the healthcare services market as a vehicle for the realisation of policy objectives and therefore different estimations of the utility of market engagement with other healthcare systems.

For the China state, the employment of domestic and international market mechanisms in pursuit of its ambitious health policy goals is seen as a pragmatic response to the scale of the demands it faces in this policy domain, as in others. Since the 1980s, levels of public dissatisfaction with the government’s introduction of ‘user pays’ financing had increased, exacerbated by difficulties in accessing basic healthcare services and an impoverishment of healthcare provision [7, 60]. Spurred by the 2003 outbreak of Severe Acute Respiratory Syndrome (SARS) and the prospect of social conflict, the Chinese government moved to develop a comprehensive strategy for its healthcare system. Social stability required both substantial health care reform and a very large increase in health insurance support [117]. Formal government recognition of the problem and its statement of strategic intent came in 2009 with the launching of *Healthy China 2020* - a political commitment to establishing an accessible, affordable, and efficient health system for all by 2020 – subsequently reinforced and expanded in 2016 with *Healthy China 2030*.

The first stage in the reform programme focused on expanding primary care, scaling up government expenditure on healthcare, and the expansion of a social health insurance model for healthcare financing [103]. Thus whilst in 2002 less than 10% of rural populations and even fewer rural-urban migrants (who do not have hukou benefits in the urban areas where they reside) had basic medical insurance coverage, by 2017 the combined figure had risen to 95%, accompanied with an increasing level of average per capita financing [61]. However, it needs to be born in mind that total health expenditure as a % of Gross Domestic Product (GDP) fell from 4.5% in 2000 to 3.7% in 2007, before rising to 5.3% in 2018, still far below European Union (EU), Organisation for Economic Cooperation and Development (OECD) and United States (US) levels [111]. What is clear is that Chinese healthcare users remained unimpressed by the coverage and quality of public health insurance with more than one-third reporting it unsatisfactory in a 2016 survey ([29]: 2; see also [113]). Since this has meant there is unmet demand and since private health insurance is a growing but limited part (3.6%) of China’s health expenditure (despite state tax incentives designed to promote its use) individual self-pay has continued to fill this funding gap and currently constitutes 35% of the total health expenditure [110]. Meanwhile, the substantial presence of unmet demand in the international healthcare market from the more affluent sector of China’s health consumers (as opposed to unmet demand from poor farmers and urban residents) is evidenced by the US$ 10 billion spent on medical tourism in 2015, a growth rate of 500% over the previous 3 years [53]. Here the established market pattern is that 95% of these healthcare consumers went to other Asian countries rather than to Europe or the US [34]. At the same time, state support for external engagement in this policy field, as in the domain of economic policy in general, encourages healthcare officials at regional and local level to send delegations to the US and the UK to build links and explore the potential for accessing treatment and professional education (see eg [18, 99]). Meanwhile, the government has launched an online platform to help its citizens travel to the US, Japan, and South Korea for care [87].

A significant ideological shift had already taken place prior to *Healthy China 2020*. With market adaptations to opportunities offered by the rise of the middle-class healthcare consumer and roots in economic and social reforms introduced since 1979, but crystallising in a set of 2009 health system reforms and the 12th Five Year Plan (2011–15), the China state addressed the supply side response to the rapidly expanding demand through a series of measures designed to enhance provider capacity and efficiency through the use of privatisation and market competition [79]. Public hospitals were required to be self-financing through the collection of patient fees for examinations and treatments [94, 108]. Regulations were introduced to encourage both domestic and international private investment in new hospitals, backed by tax incentives (see eg State Development and Reform Commission et al. 2010 [92, 93]). This approach was taken further with the permitting of 100% foreign ownership and management of private hospitals, replacing the previous requirement of a minimum of 30% Chinese ownership, bringing together the two market strands of investment and ownership [27, 73]. At the same time, in support of this supply side strategy the healthcare labour market was freed up to allow doctors working at public hospitals to practice at private facilities as well so that new private hospitals could compete with one another (and with public providers) for the medical professionals they required [72]. More recently, a series of government measures have been introduced designed to increase the flexibility of public-private arrangements; for example, private healthcare institutions are permitted to join consortia led by large public tertiary hospitals; the encouragement of ‘social capital’ (the term used to refer to domestic private investment) in the development of chained, grouped and large-scale clinics; expansion of the range of private clinics allowed to engage with fee-based public medical insurance; and permissions for healthcare institutions to provide and administer their own medical and social insurance schemes [37, 90, 91, 95].

The impact of these demand and supply side reforms has been to create a mixed economy where the state-sponsored market dynamic is rapidly reshaping China’s healthcare system. Between 2011 and 2017 the number of private hospitals doubled to 19,759 hospitals - 64% of the total. In the same period, private hospital beds grew at a CAGR of 31%, compared to 6% for public hospitals [19]. That process of rapid change propelled by the private sector has continued so that in 2020 there were 23,524 private hospitals and 11,870 public hospitals with the projection that in the following years the private hospital sector would continue to increase [74]. However, whilst the private sector’s institutional presence is clearly expanding this is likely to consist of smaller hospitals and medical centres with the large public hospitals continuing to provide the majority of beds. At the end of 2020, there were 7.131 million hospital beds in China (up 264,000 beds compared to 2019), of which public hospitals accounted for 71.4% (up 115,000 beds from last year), and private hospitals 28.6% (up 150,000 beds from last year) [75].

If the approach of the Chinese state to its healthcare system is characterised by the pragmatic search for workable models where the use of demand and supply side market mechanisms coupled with private investment are seen to provide a large part of the answer, that of England’s National Health Service (NHS) is informed by a commitment to a wholly government funded health service, comprehensive in scope and free at the point of delivery within which markets play a minor role [39]. On the demand side, although the internal market reforms of England’s NHS from the 1990s onwards emphasised the central role of the patient in decisions about healthcare service delivery, this emphasis was manifest in the inclusion of ‘the healthcare consumer’ through bureaucratic means rather than through the transfer to them of market purchasing choice, money and power [86]. Control of the demand side of the healthcare system remained firmly in the hands of state commissioning agencies of one kind or another. At the same time there has, logically, been no state encouragement for the private healthcare consumer through incentives for the private health insurance sector. Hence the individual purchase of private medical insurance in the UK has been in long-term decline since the 1990s ([20]: 2). In the UK private health expenditure in 2017 comprised 20.6% of the overall total spend, with self-pay expenditure contributing 15.9% [109, 110]. The extent of this out- of- pocket expenditure is in part a response to waiting lists for NHS care. For example, the increases in self-pay for private services in acute medical care by an average of 7.4% per annum in the 2014–2018 period have been attributed to these [56]. Longer waiting lists as the NHS recovers from the impact of Covid 19 period have also brought about predictions of a further expansion of the self-pay market in the current period [57].

Whilst the UK state has consistently worked to ensure that the demand side of the UK healthcare system remains largely impervious to any substantial market dynamic, its approach to the supply side of the English NHS has employed a range of market-oriented policies such as the private finance initiative, the development of independent sector treatment centres, new contractual arrangements for delivery of primary care in which general practitioners contract to private companies and not to the state, and the generalised outsourcing of support services [70]. In common with healthcare systems in many high-income countries, the internal market reforms embraced the ‘new public management’ (NPM) approach drawing on private sector business models of managerialism in the introduction of market-oriented plurality and competition in the provision of public healthcare services: the assumption being that this would lead to improved efficiency and quality of service delivery [43]. A key part of the reforms was the establishment of decentralised NHS Foundation Trusts with additional freedoms to own their land, borrow from public or private sectors, run joint ventures with the independent sector, and make surpluses and losses. Treasury rules were changed to enable Trusts to utilise the commercial potential of their physical and non-physical assets through the selling of existing goods and services, developing new goods and services from existing assets, licensing and leasing arrangements, and sponsorship activities [96].

Initially Trusts were required to limit the proportion of their private income to the level it had been in 2006. For many this was zero, the average across England was 2%, but proportions were considerably higher in teaching hospitals, especially in London [56]. Importantly, the Health and Social Care Act 2012 changed this by allowing Trusts in England to increase their private patient income to up to 49% of their total income. Advocates of this legislation foresaw opportunities to access the international healthcare services market and to secure greater numbers of patients travelling from overseas for treatment as part of such wider commercial development ([65]: 341). Pragmatists saw it as a potential means for offsetting the continuing financial squeeze on Trust finances [71]. Here the engagement of English healthcare sector organisations in activities beyond domestic provision has been actively encouraged by government agencies, though with limited success [104]. The now-defunct NHS Overseas Enterprise (NHSOE) was at the forefront of early efforts for cross-border exchanges in the 1990s [5]. More recently, intermediaries such as British embassies and governmental bodies such as NHS Global (2010–2012) and Healthcare UK (2013-present) (the latter is described as ‘part of the Department of Health and Social Care, the Department for International Trade, the NHS England and the NHS Improvement’ [42]) have been tasked with promoting and facilitating networks by providing technical assistance and organising trade visits to countries such as China.

In practice, this commercialisation meant the acquisition by NHS organisations of the managerial skills necessary to support the use of private sector strategies, approaches and market-based transactions [65]. However, there are strong indications that these have had limited managerial impact. Even a cursory reflection on the debate surrounding ‘privatisation’ and the NHS would suggest that the importation of market and private sector concepts and techniques into its organisation has not been entirely unquestioned (see eg [52, 83]). It is notable that the 2019 *NHS long term plan* for England does not once mention ‘income generation’.

As a policy intervention designed to stimulate greater use of the market, the 2012 increase in the threshold of private income to 49% of total Trust income produced little overall effect. Between 2012/13 and 2015/16 NHS income from private patients increased from £511 million to £596 million – as a proportion of NHS expenditure an increase from 0.63 to 0.74%. As a proportion of completed NHS treatments, private patients rose from 0.49 to 0.5% over the same period. Ten private patient units – mainly based in London NHS hospitals – accounted for nearly 60% of the £596 million generated. For four of these hospitals, private patient income made up more than 10% of their income with about a quarter of patients coming from overseas [12]. For example, London’s Royal Marsden NHS Foundation Trust’s income from private patients in the year up to March 2020 accounted for 36% of the Trust’s patient revenue and 29% of total revenues and a new cancer treatment centre is set to open near Harley Street [81]. Meanwhile in the private hospital sector, overseas patients accounted for about 2.8% of revenue though, again, a much higher percentage of 23% of private hospital revenue in London [20]. In this respect the UK’s interaction with the medical tourism market can be described as predominantly an elite hospital activity in the south of the country. It is mostly those hospitals with established international clinical networks and experience of private sector work which have the capacity to access the potential demand from China.

The China and the UK central governments have different approaches to the use of the healthcare services market and, it can therefore be anticipated, to the construction of market relationships between the two healthcare systems. Whilst China is actively seeking to harness national and international market mechanisms to achieve its ambitious healthcare strategy, the UK’s approach is more ambiguous. On the one hand, the NHS has a traditional commitment to a healthcare system where the market plays, at best, a subordinate role. On the other, parts of the UK central state such as the Department for International Trade are keen to promote the NHS as a vehicle for expanding overseas trade. With its health expenditure as a proportion of GDP presently standing at only 5.1% compared to the UK’s proportion of 9.6%, the OECD’s 12.5%, the world’s 9.9% and the United States’ 17.1%, and an ambitious set of healthcare policies in place, China has the potential to develop its healthcare system considerably as it seeks to deliver for its population [107]. As it seeks to do so, this demand for the knowledge and skills to achieve its ambitions may represent buying power to which the UK healthcare system could provide at least some of the supply response as China engages with the world healthcare economy.

## Method

How far and in what ways has this potential market relationship between the two healthcare systems been realised and what has facilitated these relations? In addressing these questions, data were gathered using two interrelated methods. First, in a broad scoping exercise, internet data were collected to provide a descriptive profile of the English healthcare institutions with agreements to work on a commercial basis with Chinese counterparts. This method identified 23 cases. As the 23 agreements are commercially sensitive in nature, the information made available by the involved parties tends to be limited to announcements on the form and nature of the agreement. In some cases the value and length of contracts has been released publicly and is reported here, however such cases are in a small minority. Our data are unlikely to include all such England-China relationships, since it is probable that some relationships have not been promoted in online public fora, and the activities set out in some agreements may not (yet) have materialised. Rather we provide an illustration of the range of relationships being developed.

Data on the 23 cases were collected through detailed examination of online sources. This began with a review of promotional materials and annual reports produced by Healthcare UK. Our search then expanded using the institutional websites and promotional materials of public and private healthcare organisations in England. We also conducted a search of press media coverage using the names of these organisations in English language and Chinese search engines, identifying an additional 804 sources which were stored in case-specific notebooks in EverNote. In order to capture emerging information on new agreements being signed after the research commenced, one of the authors set up Google Alerts using the names of English healthcare organisations known to be active in this area, combined (using Boolean operators) with ‘China’. The retrieved information was synthesised in MS Word to produce a set of 23 profiles that detail each case, the stated aims for involved parties, and the commercial relationships attempted and achieved.

Second, these descriptive profile data were supplemented with data from interviews with 33 respondents based in the UK (9) and China (24) who have detailed knowledge of UK-China trade in health-related services. Here the objective was to probe beyond the descriptive profile, to build on initial understandings, and to provide qualitative insights into how the agreements were constructed and the role of networks and brokers in that process. To that end, the interviewees approached included senior managers or clinicians in government agencies, public and private healthcare providers, industry associations, investment firms, insurance companies, consultancies and business media. Interviews were conducted by phone or in-person by at least one of the co-authors during Nov 2019 – Mar 2021, and questions covered the scale and form of UK-China trading in health-related services, experiences developing these relations, and general perceptions of working in the context of UK-China trade. The researcher(s) took detailed notes during and following the interview. Ethics approval for the research was provided by King's College London Ethics Committee.

For the analysis, all the co-authors read through the internet-generated case notes in an inductive process, seeking to identify patterns and themes in the form and function of England-China healthcare export relations together with the role of networks and brokers in the construction of those relations. A coding frame with an initial set of categories of healthcare services commercial relationships was created from the internet data, tested against a sub-set of that data, and then refined in the light of two criteria: the categories’ ability to act (a) as a discriminating conceptual tool and (b) as a useful analytical node for the exploration of the role of networks and brokers. Although we initially considered the data with reference to the General Agreement on Trade and Services (GATS) categories, in order to meet our criteria we found we needed to further distinguish and categorise within modes, and therefore grouped cases according to an alternative set of three categories representing healthcare services based on different types of knowledge: direct and indirect patient care (clinical knowledge), professional education (educational knowledge), and hospital and healthcare service development (managerial and investment knowledge). Within each category we examined the presence of clinical, state and private networks and third-party brokers. Thus armed with an initial interpretation of the commercial relationships as a result of this first stage, the interview data were then interrogated using a coding framework designed (a) to test the accuracy of this interpretation and (b) further explore and refine our understanding of the issues regarding the causal processes at work in the construction of the relationships. Although this framework used the basic format of the first stage analysis, this was applied in a way which encouraged the creation of sub-categories in pursuit of greater flexibility and hopefully a more nuanced interpretation of the construction of the commercial relationships. Where possible, the overall findings from this two-stage approach have been validated through consultation with representatives from organisations and experts with knowledge of these markets.

## Results

Little financial data exists on the overall commercial relationship between the healthcare systems of China and England. Table 1 summarises what we have found regarding the position of the UK (not just England) as a supplier of healthcare services in the international market. This centralised data does not permit disaggregation by country of the UK but it seems clear from other sources that the vast majority of this activity is by organisations based in England. Even with this very limited data source culled from the annual reports of Healthcare UK between 2014-15 and 2017–18, it is apparent that from the UK perspective China occupies an overwhelmingly dominant position in that market. In the 4 year period covered by the reports, the UK’s healthcare services business with China amounted to GBP 3978 million – 70% of its total international business in this field. The data also shows that, within this, the predominant market sub-sectors are ‘infrastructure’ (our ‘hospital and healthcare service development’) and ‘clinical services’ (our ‘patient care’).

**Table 1:** Healthcare UK: reported business wins (2013–2016) and export wins (since 2017) (GBP million)

2014–15		2015–16		2016–17^a^		2017–18	
Total: 749 (from 26 projects)		Total: 3700 (from 67 projects)		Total: 540 (unknown number of projects)		Total: 700 (from 101 projects)	
**By country**:		**By country**:		**By region:**		**By region:**	
China	584.6	China	2190	Middle-East	124	China	363
UAE	90	Saudi Arabia	697	China	120	Middle-East	186
Brazil	47.7	Brazil	323	India	47	Latin America	71
Kuwait	10	UAE	316			India	60
India	2.1	India	126			Central and Eastern Europe	32
Libya	1.2	Qatar	60				
Hong Kong	1						
**By area of work**:		**By area of work**:				**By area of work**:	
Infrastructure	590.4	Infrastructure	2665			Clinical services	200
Clinical services	110.5	Clinical services	899			Infrastructure	180
Digital health	27.9	Digital health	142			Educ and training	50
Educ and training	20.1	Educ and training	17			Digital health	40

^a^ Figures for this column have been estimated based on year-on-year increases reported in the 2017/18 annual review, and even then only a regional breakdown was reported, not area of work. ‘Business wins’ and ‘export wins’ are terms used by the UK Government: ‘business win’ refers to the total value of a contract signed by a UK company; ‘export win’ refers to the proportion of a contract’s value that would accrue to UK companies (and not, for example, to partner companies based in-country).

*Source:* Healthcare UK Annual Reports (2014–2018).

Turning now to the findings from our institutional analysis, Table 2 shows how the market demand from China for the knowledge resources to support its healthcare system development and enhance the amount and quality of its domestic healthcare service provision are distributed across the three sub-sectors of supply for each English healthcare institution. In a significant minority of cases the Chinese demand explicitly forms part of a larger strategic objective where the knowledge is intended to act as the platform for the Chinese institution itself to enter the international healthcare market and become an international provider (‘Internationalisation’ in Table 2).

**Table 2:** England-China healthcare services commercial relationships

***England Institution***	***China Partner (public/ private)***	***Type of Service Agreement***	***Broker Partner***
Alder Hey Children’s NHS Foundation Trust	Public hospital: Chengdu Women and Children’s Central Hospital	Prof education Patient care (bidirectional referral system)	Private Company: Beijing Huatong Guokang Foundation
University Hospitals Sussex NHS Foundation Trust	Public hospital: The Second Hospital of Shandong University	Prof education	None used
Bupa Healthcare	Private Company: Shanghai Alltrust Insurance Company	Private insurance	None used
Cambridge University Hospital NHS Foundation Trust	Public hospital: Hunan Provincial People’s Hospital	Patient care (consultation in elder care)	None used
	Public hospital: Beijing China-Japan Friendship Hospital	Prof Education	None used
	State-owned enterprise: Shanghai Shenkang Hospital Management Company	Prof Education	None used
	Public hospital:: China Pharmaceutical University Hospital	Prof Education	None used
	Public hospital: West China Hospital of Sichuan University	Prof Education	None used
	Public hospital: Changzhou First People’s Hospital	Prof education and accreditation	Private company: UKeMED
	Public hospital: West China Hospital	Prof education	Private company: UKeMED
	Public hospital: Chongqing Renji Hospital	Prof education Internationalisation	Private company: UKeMED
	Public hospital: Zhenjiang First Hospital	Patient care (consultations)	Private company: UKeMED
	Public hospital: Xinghua People’s Hospital	Patient care (consultations)	Private company: UKeMED
	Public hospital: Jingjian People’s Hospital	Patient care (consultations)	Private company: UKeMED
Circle Health	Public hospital: Shanghai Ruijin Hospital	Patient care (consultations)	None used
GDK Healthcare	Private company: Beijing Huatong Guokang Foundation	Prof education	Private company: Beijing Huatong Guokang Foundation
	Public hospital: Ningbo Second Hospital	Prof education (mental health) Internationalisation	None used
	Public hospital: Chengdu Women and Children’s Central Hospital	Prof education (paediatrics)	None used
	Government: Health Commission of Chengdu	Prof education (GP training)	None used
	Public hospital: Ningbo Mental Health Hospital	Prof education and accreditation	None used
Great Ormond Street Hospital for Children NHS Foundation Trust	Public hospital: Hunan Children’s Hospital	Prof education	None used
	Public hospital: Beijing Children’s Hospital Group	Prof education	None used
	Private company: Saint Lucia Consulting	Patient care	Private company: Saint Lucia Consulting
Guy’s and St Thomas’ NHS Foundation Trust	Government: Nanjing Government	Hospital service development Nanjing	None used
Heythorp Healthcare China	Private company: CITI Group	Healthcare service development Kunming (elderly)	None used
	Private Company: Shanghai Soyoung Management Co. Ltd	Healthcare service development Shanghai (elderly)	None used
Imperial College Healthcare NHS Trust	Government: Beijing Health and Family Planning Committee	Patient care	None used
	Public hospital: Yunnan First People’s Hospital	Prof education	None used
	Public hospital: Sichuan’s Public Hospitals	Prof education	Private company: Beijing Huatong Guokang Foundation
	Public hospital: Shandong’s Public Hospitals	Prof education	None used
	Private company: St Lucia Consulting	Patient care	Private company: St Lucia Consulting
International Hospital Group (Yingci Healthcare)	State-owned enterprise: Guangzhou International Medical Port	Hospital service development Guangzhou Internationalisation	None used
	Private company: Hangzhou Keyi Real Estate Development	Hospital service development Hangzhou Internationalisation	None used
	Private company: Qingdao-Wanda Group International Hospital	Hospital service development Qingdao Internationalisation	None used
King’s College Hospital NHS Foundation Trust	Private company: Hongtai Consulting	Patient care	None used
	Government: Beijing Health and Family Planning Committee	Patient care	None used
	Private company: Hangzhou Healthcare Management	Patient care	Private company: Hangzhou Healthcare Management
	Public hospital: Oncology Hospital Affiliated with Tianjing Medical University	Prof education	None used
	Public hospital: Sichuan West China Hospital	Prof education	None used
	Public hospital: Peking University Sixth Hospital	Prof education	None used
Northumbria Healthcare NHS Foundation Trust	Private company: Rongqiao Group	Hospital service development Fujian Internationalisation	None used
	Public hospital: Hunan Xiangya Hospital	Prof education (nursing)	None used
	Private company: Inteli Healthcare	Patient care	Private company: Inteli Healthcare
Oxford University Hospital NHS Foundation Trust	Public hospital: The First Affiliated Hospital of Xiamen University	Patient care (Consultation)	None used
Royal Brompton and Harefield NHS Foundation Trust	Private company: Guangzhou Hongtai Consulting	Patient care (heart disease)	Private company: Guangzhou Hongtai Consulting
	Private company: Hangzhou Wuzhou Healthcare Management	Patient care (heart disease)	Private company: Hangzhou Wuzhou Healthcare Management
	Private company: Inteli Healthcare	Patient care	Private company: Inteli Healthcare
	Private company: St Lucia Consulting	Patient care	Private company: St Lucia Consulting
	Private company: GDK Healthcare	Patient care	Private company: GDK Healthcare
Royal Free London NHS Foundation Trust	Private company: Guangdong E-tech Specialist Centre	Patient care (consultation) Internationalisation	None used
	Private hospital: Beijing Henghe Hospital	Nurse education and accreditation	None used
	Government: Health and Family Planning Commission, Zhejiang and Shandong Provinces	Prof education (clinicians)	None used
	Private company: Lingchuang Cosmetic hospitals, Aimei Group	Prof education (clinicians – cosmetic surgery) Internationalisation	Private company: MedEther Group
The London Clinic	Private hospital: Shanghai International Medical Centre	Patient care Internationalisation	None used
The Royal Marsden NHS Foundation Trust	Private company: Beijing Weirenweiye International Medical Research Centre	Prof education	None used
	Public institute: Heilongjiang Medical Association	Prof education	None used
	Private company: Beijing Huatong Guokang Foundation	Prof education	Private company: Beijing Huatong Guokang Foundation
	Private company: Auro Medical Consulting	Patient care	Private company: Auro Medical Consulting
	Private company: St Lucia Consulting	Patient care	Private company: St Lucia Consulting
	Private company: GDK Healthcare	Patient care	Private company: GDK Healthcare
	Private company: Inteli Healthcare	Patient care	Private company: Inteli Healthcare
	Private company: Union Med	Patient care	Private company: Union Med
Sinophi Healthcare Ltd	Public hospital: Jiangsu Huai’an’s First People’s Hospital, Jiangsu	Hospital service development (oncology)	None used
	Government: Huai’an Government	Hospital service development (oncology)	None used
	Public hospital: Shandong Guanxian Central Hospital, Shangdong	Healthcare service development (elderly)	None used
	Private company: Jiangsu Tianchuan Lake Company, Nanjing	Healthcare service development (elderly)	None used
University College London Hospital NHS Foundation Trust	Public hospital: Hunan Oncology Hospital	Prof education	None used
	Public hospital: Seventh Affiliated Hospital of Sun Yat-Sen University	Prof education	None used
	Private company: Beijing Huatong Guokang Foundation	Prof education	Private company: Beijing Huatong Guokang Foundation
	Public hospital: Shenzhen People’s Hospital	Prof education	None used
	Public institute: Ningbo Medical University College	Prof education	None used
	Public hospital: Leshan Hospital	Prof education	None used
	Private company: GDK Healthcare	Prof education	Private company: GDK Healthcare
University Hospitals Birmingham NHS Foundation Trust	Guangzhou Municipal Health Commission	Prof education (GP Training)	None used
	Public hospital: First Affiliated Hospital of Sun Yat University	Prof education Patient care	None used
	Private Hospital: Guiqian International Hospital	Prof education Internationalisation	None used
	Private company: Sino–British (Guangdong) International Medicity	Hospital service development Internationalisation	None used
	Private company: China Health and Elder Care Group	Prof education (elder care)	None used
	Private company: Soufine Healthcare	Prof education (management)	None used
St George’s University Hospital NHS Foundation Trust	Public Hospital: Cangzhou People’s Hospital	Healthcare service development and prof education and training	None used
	Government: Shandong Provincial Health Commission	Prof education: Six-week Observership at the St George’s Hospital	None used
Liverpool Women’s NHS Foundation Trust	Private company: Xi’an Taikang Hospital Management	Hospital service development Internationalisation	None used

In response to this demand, most of the NHS Trusts who are involved in such activity focus on supplying a mix of patient care and professional education, drawing on the knowledge assets embedded in their existing activities and specialties and adapting them to the needs of the China market. It is then left to the private sector institutions, in particular the International Hospitals Group (IHG), Heythorp and Sinophi, to exploit the potential of the hospital and healthcare service development sub-sector where the contract value is much larger and the acquisition of the management and investment knowledge required is beyond the normal capacities of NHS Trusts. Of the 16 Trusts identified in the mapping, only Liverpool Women’s NHS Foundation Trust, Northumbria Healthcare NHS Foundation Trust, St George’s University Hospital NHS Foundation Trust, and University Hospitals Birmingham NHS Foundation Trust appear to have had agreements in this market sub-sector.

Given the ambition of China’s healthcare reforms and its experience of sourcing healthcare expertise through the international market, the UK supply of clinical, educational, and managerial and investment knowledge has to bear the weight of China’s high market expectations, influenced as these are by China’s established relationship with the US healthcare system and, to a lesser extent, that of Japan. Our interviewees often noted the development of the former relationship over the past twenty years with Chinese government officials and clinicians receiving their training in the US, returning to China and then later in their careers are ‘naturally inclined’ to facilitate partnerships with US healthcare institutions (Interview 18). A senior manager in one UK-based private provider observed that ‘in general Chinese hospitals cooperate more with hospitals from the US’ and ‘very little’ with those from the UK because the US entered the Chinese market earlier and so has ‘a longer history, more cooperation and greater influence’ (Interview 20). Reputation, high clinical standards, higher treatment success rates than in China, access to leading technologies, and the ability to be included in the clinical trials of the latest drugs are the reasons quoted by a China-based broker company’s manager for advising patients to travel to the US as the first choice for treatment (Interview 31).

Given this context, if UK institutions are to be internationally competitive on a sustained basis in any area of healthcare market construction, they must have particular characteristics to give them a market advantage. It is, then, no coincidence that all the NHS Trusts with market relationships with China are high status with a specialist reputation, deemed capable of facilitating the formation of a partnership and establishing the trust necessary for an enduring relationship and legitimising the quality of the knowledge assets which are to be internationally traded through it. For example, on its website NHS Northumbria International Alliance (2020) - a partnership between Northumberland NHS Foundation Trust and Northumberland County Council supported by agreements with the Christie NHS Foundation Trust (the largest single-site cancer centre in Europe) and the Liverpool Heart and Chest NHS Foundation Trust - stated:

From training through to design and build, the services that NHS Northumbria International Alliance provide are delivered by only the elite of the NHS, i.e. only those who have achieved and maintain ‘Outstanding’ standards by an independent body (Care Quality Commission -CQC) [76].

The UK’s private sector organisations with China partnerships, dependent as they are on the expertise of NHS employed consultants on which the strength of their knowledge market position relies, are also concerned that these should be drawn from Trusts in England that have an internationally marketable reputation. This is reflected in the leading NHS Trusts with which they choose to publicly associate themselves. For example, GDK’s list includes Cambridge University Hospital NHS Foundation Trust, Imperial College Healthcare NHS Trust, Guy’s and St Thomas’ NHS Foundation Trust and Great Ormond Street for Children NHS Foundation Trust as its NHS partners and the IHG, The Christie NHS Foundation Trust, South Tees Hospitals NHS Foundation Trust and Royal Free London NHS Foundation Trust [32, 48].

From the Chinese perspective, status provides a ready indicator of the quality of the knowledge service they choose to purchase. Hunan Oncology Hospital, for example, reports that its choice of University College London Hospital NHS Foundation Trust as a partner is based on the Hospital’s affiliation with University College London which, it notes approvingly, is a top university with a global reputation manifest in its membership of the G5 grouping of elite universities together with the University of Cambridge, the University of Oxford, Imperial College London and the London School of Economics and Political Science [30]. Reputational transfer of this kind improves the China partner’s own market position and the prices it can charge its customers. When the external recognition of high-quality contract delivery is achieved, it is important that this, and the reputational and market enhancement it confers, is publicly noted. Hence when Alder Hey Children’s NHS Foundation Trust’s (AHCH) observership programme for surgeons, nurses and pharmacists from the Beijing Huatong Guokang Foundation gained the UK Department of International Trade’s Greater China Educational Link Award in February 2019, the Head of Alder Hey’s Academy emphasised: ‘This award is imperative to the future success of international business opportunities for Alder Hey Academy and Alder Hey Children’s Hospital both in China and worldwide’ [2].

In order that the value of the knowledge service should be fully realised, Chinese expectations met and a secure market relationship established, quality assurance through evaluation or accreditation is a common project component. This is particularly important to a China healthcare services sector where, as a report from the professional services network KPMG points out, standards and regulation are underdeveloped resulting ‘in a market that lacks order and sophistication for an industry which is experiencing a fast pace and strong momentum of development’ ([55]: 59). As part of the agreement package, therefore, UK partners often provide the standards by which the value of their knowledge asset transfer is to be judged, as a commodity in their own right. This is perhaps most evident in the professional education sub-sector of the market. For example, in its agreement with Ningbo Mental Health Hospital to support the establishment of an international psychiatrist training base for South-East Asia, GDK states it will draw on resources from British medical schools, clinics and continuing education to support the work of the Medical Training Expert Group tasked as part of the deal with the formulation of norms, standards and certification for medical education [77]. And as part of their agreement, Cambridge University Hospitals NHS Foundation Trust and Changzhou First People’s Hospital planned to issue joint qualification certificates for medical personnel who are trained in general practice, resident doctor standardisation and quality management standards for clinical drug trials in the Changzhou Hospital [13].

Again, in the healthcare service development sub-sector of the market, the UK partner is expected to establish and implement quality standards. Thus as part of the agreements for the building of the Wanda-IHG International Hospital in Qingdao and subsequently hospitals in Shanghai and Chengdu, it is IHG which has the responsibility for ensuring that the hospitals are granted approval from the Joint Commission International (JCI) on Accreditation of Healthcare Organizations [33]. Similarly, the agreement between Northumbria Healthcare NHS Foundation Trust and the Rongqiao Group to build Ronqiao International Hospital is underpinned by the claim that it is the first hospital in China to be constructed using the NHS system of CQC standards [40].

## Discussion

The translation of these three types of UK healthcare knowledge assets (clinical, educational and managerial/investment) into market value, their incorporation in a rules-based agreement, and the evaluation and maintenance of the knowledge service over time, requires the painstaking construction of durable networks. Many NHS Trusts are managerially ill-equipped for this task. Lunt et al.’s [65] research on medical tourism shows how in this market Trusts can be ‘hampered by not having a commercial team, alongside limited marketing budgets, and also [constrained by] potential internal perceptions about private work’ plus the inability to handle commercial uncertainty – with the private sector seen by comparison to have the skills and the advantage (p342). They continued, ‘the NHS orientation was a passive one whereas private and international activity may involve more marketing and proactivity engaging overseas markets’ (p342). Such proactivity entails the construction of robust social networks through the use of cultural resources and international connections available mainly to the leading specialist NHS Trusts and private healthcare organisations.

The construction of the networks necessary to organise and maintain service agreements for knowledge transfer between UK and Chinese institutions varies across the three market sub-sectors of patient care, professional education, and hospital development, but with certain common features. Common to all is the formal expression of mutual trust at an early stage in the relationship through a memorandum of understanding or cooperation or, on occasions, a declaration of a ‘strategic agreement’. Typically, in the case of NHS Trusts, such agreements build on prior networks of clinicians, established through their international professional work, and contacts established by an institution’s commercial team. For example, the professional education agreement between Cambridge University Hospital NHS Trust and Shanghai Shenkang Hospital Management Company (Table 2) for the training of senior doctors, managers and vice-presidents from Shanghai’s level-A tertiary hospitals was initiated and facilitated by a Chinese professor of medicine at the University using his existing professional networks (Interview 19). Similarly, that between University Hospitals Birmingham NHS Foundation Trust and Guangzhou Municipal Health Commission (Table 2) for GP training was also the result of entrepreneurial activity by a Chinese professor at the University who saw the opportunity, established the connections between the two institutions, and identified the administrative requirements to make the commercial agreement possible (Interview 25). Often the occasion confirming the agreement is described as a ‘signing ceremony’ indicating the important ritual aspect and permanence of the trust relationship, such as that between Great Ormond Street Hospital for Children NHS Foundation Trust and Hunan Children’s Hospital, Cambridge University Hospital NHS Foundation Trust and Chongqing Renji Hospital, and Royal Free London NHS Foundation Trust and the Aimei Group [1, 16, 45].

For the large agreements where investment companies are involved and the financial and political stakes are much higher, a state presence is customary to enhance and, hopefully, ensure the public significance, market visibility and legitimation of the relationship. Unsurprisingly, given their established presence in the international healthcare service development sub-sector and investment expertise and networks, it is the private sector which can most readily call upon its China and UK state networks for support. For example, the £92 million partnership agreement between Yingci Healthcare (a subsidiary of IHG) and Hangzhou Keyi Real Estate Development to build the Yingci-Keyi International Hospital in Hangzhou’s Xiaoshan district was signed in the presence of Liam Fox, Secretary of UK Trade and Investment [100], and the IHG-Wanda Group agreement to build the Qingdao-IHG International Hospital witnessed by Jeremy Hunt, UK Secretary of State for Health and Social Care [102]. Such high-level networks are also cultivated by IHG with the China state. Chester King, chairman of IHG Asia, for example thought it worthy of comment in 2015 that: ‘In May IHG was accepted as the only foreign member of the Strategic Alliance of the Healthcare Industry of China (SAMHIC) and nominated as Head of its International Affairs Committee’ [17].

Sinophi Healthcare, a British company providing investment and management services for Chinese hospitals, has enjoyed a particularly close relationship with the UK state’s Department of Health and Social Care, the UK Trade and Investment Department (now Department for International Trade), the NHS, and Healthcare UK [9]. During China’s President Xi Jinping’s state visit to the UK in October 2015, Sinophi Healthcare signed seven cooperation agreements with China’s hospitals and private investors, with a total value of £700 million [97]. Prior to this in 2013 during the visit to China of David Cameron, the British Prime Minister, the company signed a cooperation agreement worth £120 million with Huai’an’s First People’s Hospital, jointly to establish an oncology hospital with 1000 beds [80]. Officials from both the UK Department of Trade and Investment and the Huai’an municipal government attended the signing ceremony [44]. High status legitimation of both a specific and general nature accompanied the event with David Cameron commenting: ‘This deal highlights the enormous opportunity that the Chinese healthcare market presents for British healthcare firms - set to grow by US$ 400 billion by 2017. I hope we will see many more partnerships and deals like this for British businesses in this market’ [106]. Jeremy Hunt, Health Secretary, and also on the visit, observed: ‘These agreements will see UK firms exporting their expertise to China – building their businesses and investing in Chinese healthcare. Both countries will benefit from these new relationships and better trade links.’ [106].

Orbiting the established international healthcare providers are a range of specialist brokerage firms concentrating on the construction of market relationships in particular sub-sectors of the market, drawing on their own dedicated networks with NHS Trusts and Chinese clients. For this the private sector company GDK Healthcare, for example, provides a brokerage function in the sub-sector of patient care for the Royal Brompton Hospital and Royal Marsden Hospital and in the professional education sub-sector for University College Hospital (Table 2). The role of brokers in facilitating the patient care sub-sector of the market is particularly well established, and this is reflected in their prominent position in the China-UK agreements of Table 2. Some Trusts make particular use of brokers to maximise their flow of China patients for direct or indirect health care such as the Royal Marsden Hospital (five brokers) and the Royal Brompton Hospitals (five brokers) (Table 2). As with individual patient care where the broker often provides an infrastructure of travel, visas and accommodation, the added market value supplied by the professional education broker is achieved not only through the demand-supply linkage but, equally importantly, the facilitation of a technological platform of online learning and professional-student interaction necessary to activate this linkage through the flow of medical and educational knowledge from the UK supplier to the China educational consumer ([68]: [105]). Further value may accrue for the UK partner through a broker’s extended networks. Hence, the MedEther Group, a UK-based company specialising in connecting British hospitals and senior medical staff with Chinese healthcare professionals, in 2018 complemented its professional education agreement with the Royal Free Hospital with a three-way agreement with the Aimei Group, a Chinese medical cosmetology group, to work jointly to expand the medical cosmetic market in China and other Asian countries [1, 67]. On occasions the China state itself acts as a broker as in the case of the Beijing Huatong Guokang Foundation, a public foundation for helping Chinese medical workers receive overseas training, which has agreements with five NHS Trusts for the provision of professional education (Table 2).

## Conclusions

Healthcare services is an expanding international market with which national healthcare systems engage, and from which they benefit, to greater and lesser degrees. China and England have different approaches to this market. Propelled by the logic of its state-sponsored but consumer-based expansion with large private sector involvement, China is committed to exploit the international market through the creation of fresh demand-supply relationships. England’s healthcare system has the knowledge required for the supply, while such economic engagement is supported and actively encouraged by the UK’s trade related departments of state, and has been facilitated by changes in legislation. However, it is constrained by multiple domestic demands on its resources and by the limits of the NHS approach to marketisation in healthcare.

As a result, the development of China-England commercial relationships in patient care, professional education and hospital and healthcare service development has been led largely by high status NHS Trusts and private sector organisations with the entrepreneurial capacity to exploit their market position. Drawing on their established international networks of clinicians and commercial teams with experience of domestic private sector provision, these institutions have built trust-based collaborations sufficiently robust to facilitate demand-supply relationships in the international healthcare services market. Often key to the development of relations required to make commercial exchange feasible and practicable are a range of international brokers with the skills and capacity to provide the necessary linkage with individual healthcare consumers and institutional clients in China.

The centrality of networks, brokers, status and trust to the construction of these complex, knowledge-based market relationships poses some interesting policy questions for policymakers, particularly in the England case where the ideological support for activities which, one way or another, seek to generate a profit using the platform of the state funded NHS, is at best ambiguous. How can the ambition of central departments of state that the NHS should make its particular contribution to the expansion of the UK’s global trade, be reconciled with the values which inform the organisational life of England’s health professionals and managers and which centre on serving domestic healthcare needs? In a context of an increasingly resource-strained NHS, where are the resources going to come from to enable Trusts to invest in further network creation, and to fulfil the contracts which are being signed? Private healthcare sector organisations appear to have been better equipped, ideologically and practically, to take advantage of international demand, raising the question: can and should Trusts compete? And to what extent should state funded facilitators such as Healthcare UK intervene in a sector where several private brokers already exist?

China’s healthcare system, in contrast, has an established track record in terms of both engagement with the international healthcare market, particularly the US, and the use of guanxi (networks) and brokers (sometimes state sponsored) as the customary vehicles for the formation of business relationships. Its continuing state supported experiments with the use of the private sector on both the demand and the supply side of the health service equation, coupled with its success as measured in terms of the rate of expansion China’s healthcare system, suggests that this policy direction is likely to be maintained and at current rates China may soon position itself as a supplier in global healthcare markets, replicating a model used to great effect in other industries.

For the future, it would be instructive to apply the conceptual lessons of this paper regarding the operation of networks, brokers and social trust in the development of healthcare services markets to other cases. The nascent status of the kinds of global markets discussed above means that there is much to be understood in terms of range of approaches adopted by institutions and their sponsoring states operating in different contexts. For example, in the case of India-England trading, the unequivocal emphasis on the private sector by the former, and its established international presence as a provider of both patient care and professional labour, coupled with its historic relationship with the UK, offers an interesting research vehicle for the further exploration of how new healthcare services markets may be constructed. Similarly, study on the impact of the COVID-19 on trading in health-related services, including the services being traded as well as the practices involved, will provide insights into the resilience of such activities and the likely future for them as healthcare systems seek to recover from the pandemic.

## Data Availability

According to UK research councils’ Common Principles on Data Policy, data supporting this study will be openly available via the UK Data Service at https://www.ukdataservice.ac.uk/

## Abbreviations

CAGR: Compound Annual Growth Rate
CQC: Care Quality Commission
EU: European Union
GDP: Gross Domestic Product
GATS: General Agreement on Trade and Services
IHG: International Hospitals Group
JCY: Joint Commission International
NHS: National Health Service
NHSOE: NHS Overseas Enterprise
OECD: Organisation for Economic Co-operation and Development
SAMHIC: Strategic Alliance of the Healthcare Industry of China
SARS: Severe Acute Respiratory Syndrome
UK: United Kingdom
US: United States
